# The Use of Periareolar Topical Lidocaine Jelly After Needle Localization May Reduce Pain of Subsequent Lymphoscintigraphy

**DOI:** 10.7759/cureus.7861

**Published:** 2020-04-27

**Authors:** Jas Virk, Bethany Smock, Muhammad Nadeem, Celina Chung, Carolyn Maynor, David Becker-Weidman, Tony Mangano

**Affiliations:** 1 Radiology, Jacobs School of Medicine and Biomedical Sciences, Buffalo, USA; 2 Radiology, Buffalo General Medical Center, Buffalo, USA; 3 Radiology, Pinehurst Radiology, Pinehurst, USA; 4 Radiology, Buffalo General Hospital, Buffalo, USA

**Keywords:** periareolar, lidocaine, localization, pain, lymphoscintigraphy

## Abstract

Purpose

Technetium Tc-99m sulfur colloid (99mTc-SC) breast lymphoscintigraphy is commonly performed to identify the sentinel lymph node (SLN) in patients diagnosed with breast carcinoma undergoing lumpectomy. The purpose of this report is to describe how the use of 2% topical lidocaine jelly immediately after the completion of needle localization and prior to scintigraphy may substantially reduce pain associated with the injection of 99mTc-SC.

Materials and methods

This was a quality improvement project. Patients were asked to score the severity of pain associated with the periareolar 99mTc-SC injections for sentinel node lymphoscintigraphy. In order to decrease the discomfort, topical lidocaine was applied to the periareolar skin after the completion of the needle localization, but prior to transferring the patient from the mammography room to the nuclear medicine department for the 99mTc-SC injections. At the time of 99mTc-SC injection, patients were asked to score the pain of injection from 0 (none) to 10 (worst).

Results

The average pain score of the women who did not receive topical lidocaine jelly was 8 (range: 5-9). In the 10 women who received topical lidocaine jelly after needle localization, the average pain score was 2.5 (range: 1-5). Interestingly, the pain score for women who discussed the possible use of lidocaine jelly with the radiologists but still did not receive topical lidocaine jelly was also low at 6.5. For patients who received the lidocaine jelly only five minutes prior to injection, the average pain score was 6.

Conclusion

The application of lidocaine jelly after the conclusion of needle localization, with a 15-40-minute delay prior to periareolar injections with 99mTc-SC for sentinel node lymphoscintigraphy, appears to substantially reduce the pain associated with the injection of 99mTc-SC.

## Introduction

Lymphoscintigraphy is a common procedure used in oncology to identify and produce a “map” of the lymphatic system in order to identify the most likely first node of lymphatic drainage, the sentinel lymph node (SLN). The SLN is important as it is considered to be the first node to contain metastasis, and the biopsy of the node may assist in cancer staging [1]. The procedure often requires intradermal injection of a radiopharmaceutical agent, usually technetium Tc-99m sulfur colloid (99mTc-SC). 99mTc-SC breast lymphoscintigraphy is commonly performed to determine the SLN in patients diagnosed with breast carcinoma undergoing lumpectomy [2].

Unfortunately, severe pain is often reported by patients during the injection of the radiopharmaceutical, and several methods have been proposed to decrease or eliminate this pain, albeit with inconsistent results [3]. Methods used to reduce the pain include the addition of bicarbonate and/or lidocaine into the radiopharmaceuticals. The pH of slightly buffered 99mTc-SC can be 6 while that of heavily buffered 99mTc-SC can reach 8.5 [4]. Lidocaine comes in various forms and can be given intravenously, topically, or subcutaneously. The time from administration until the patient notices the numbing sensation varies by route of administration. We report on the apparent effectiveness of periareolar topical lidocaine jelly application, administered after the completion of needle localization, in reducing the pain associated with the subsequent injection of 99mTc-SC for lymphoscintigraphy.

## Materials and methods

In this quality improvement project (performed partially for maintenance of certification), we asked patients undergoing lymphoscintigraphy to score the severity of pain associated with each periareolar 99mTc-SC injections for sentinel node lymphoscintigraphy on a scale of 0-10 (0: no pain, 10: extreme pain) using the NIH Toolbox® pain-intensity measures [5]. We further subdivided the ratings as follows: 0 - no pain; 1-3 - a little pain; 4-6 - moderate pain; and 7-10 - worst pain.

After routinely recording the pain score in patients, we decided to do a pilot review of administering the leftover topical lidocaine jelly used during needle localization to the periareolar skin. This was done after the completion of the needle localization, but prior to transferring the patient from the mammography room to the nuclear medicine department for the 99mTc-SC injections. The time from administration of the topical lidocaine jelly to the injection of 99mTc-SC varied from 15 to 40 minutes. No other modifications were made to the procedure from the needle localization till surgery. If physicians added bicarbonate and/or lidocaine to the 99mTc-SC solution, this continued as before (which was the case with the vast majority). However, some physicians decided to continue with their usual methods, without the use of the topical lidocaine jelly. Another group used topical lidocaine jelly immediately prior to the injection of 99mTc-SC only. In one group of patients who received topical lidocaine jelly after needle localization, there was no migration of the radiopharmaceutical visible, and the SLN was identified during surgery with an intraoperative injection of dye.

Thus, the patients fell into five groups: (A) patients whose pain score was measured prior to the discussion about using topical lidocaine jelly; (B) patients who received topical lidocaine jelly immediately after needle localization with approximately 15-40-minute delay prior to the injection of 99mTc-SC; (C) patients who did not receive any topical lidocaine jelly prior to the injection of 99mTc-SC even though they had a discussion about using topical lidocaine; (D) patients who received topical lidocaine jelly immediately after needle localization with approximately 15-40-minute delay prior to the injection of 99mTc-SC, but in whom there was no migration of the radiopharmaceutical to a sentinel node; and (E) patients who received topical lidocaine jelly in the nuclear medicine department a few minutes prior to the injection of 99mTc-SC.

## Results

As this was only a pilot review of the pain scores kept in the department to determine if a formal study of topical lidocaine jelly should be carried out (and not a prospective study), we did not collect any demographic information. However, all patients were being evaluated for breast carcinoma. While there probably was no significant difference among patients in terms of age, race, underlying co-existing pathologies, or individual susceptibility to pain, we are not certain about it.

The pain score in the 10 patients whose pain score was measured prior to the discussion about using topical lidocaine jelly, group (A), was 8 (range: 5-9), with many describing the pain as one of the worst ever. The pain score in the 10 patients who received topical lidocaine jelly immediately after needle localization with approximately 15-40-minute delay (caused by transferring the patient from the mammography suite to the nuclear medicine suite) prior to the injection of 99mTc-SC, group (B), was 2.5 (range: 1-5). The pain score for five women who did not receive any topical lidocaine jelly prior to the injection of 99mTc-SC even though they had a discussion about using topical lidocaine, group (C), was 6.5 (range: 4-8). The pain score for four women who received topical lidocaine jelly immediately after needle localization with approximately 15-40-minute delay prior to the injection of 99mTc-SC, but in whom there was no migration of the radiopharmaceutical to a sentinel node, group (D), was 8 (range: 6-9). The pain score for two patients who received topical lidocaine jelly in the nuclear medicine department a few minutes prior to the injection of 99mTc-SC was 6 (range: 4-8) (Table 1).

**Table 1:** Groups and their respective average pain scores The pain score was rated on a scale of 0-10 (0: no pain, 10: extreme pain) TL: topical lidocaine; 99mTc-SC: technetium Tc-99m sulfur colloid

Groups	Number of patients	Pain score (average)
A (prior to TL-use discussion)	10	8.0
B (TL post-needle localization)	10	2.5
C (TL discussion, but no application)	5	6.5
D (TL post-needle localization without migration of radiopharmaceutical)	4	8.0
E (TL 5 minutes prior to injection of 99mTc-SC)	2	6.0

## Discussion

Pain is likely multifactorial since bicarbonates do not always eliminate pain; however, acidity is certainly one of the causes. It is likely that the pain reported by patients associated with periareolar intradermal injection of 99mTc-SC is due to the acidity of the agent [4]. Methods previously employed to decrease the pain experienced during lymphoscintigraphy involved injecting 99mTc-SC mixed with lidocaine, buffering the 99mTc-SC with sodium bicarbonate to decrease the agent’s pH, and reducing the number of periareolar injections.

Fetzer et al. conducted a retrospective study that found that the use of a combination of lidocaine and prilocaine topical creams prior to the injection of radiopharmaceutical helped reduce pain in patients undergoing lymphoscintigraphy [6]. However, a randomized control trial conducted by Canning et al. found no significant difference between pain scores in the group that received a topical combination of lidocaine and prilocaine and the control group [7]. Hawkins et al. found that the use of subcutaneous injection of sodium bicarbonate buffered lidocaine before the injection of the radiopharmaceutical agent reduced pain as well [8]. However, it appears that this study did not allow sufficient time for the lidocaine to induce its full anesthetic effect as the injection of the radiopharmaceutical agent followed immediately afterward. Also, care must be taken when combining lidocaine and the radiopharmaceutical agent in a syringe as there is a risk of precipitation of the agent or the dye used. However, Chandler et al. found no correlation between the use or timing of anesthetic cream and perceived pain from injection [9].

In our patient review, while not a statistically valid assessment, there was a substantial decrease in the pain patients experienced due to periareolar intradermal injection of 99mTc-SC for sentinel node lymphoscintigraphy when the topical lidocaine was administered to the periareolar skin immediately after needle localization (group B) compared to the pain score revealed by patients prior to this pilot (2 vs. 8, Table 1). The reduction in pain seemed to be greater than that associated with administering the lidocaine in the nuclear medicine department five minutes prior to the injection (group E, 2.5 vs. 6, Table 1), and this suggests that the duration of time between the administration of lidocaine and the injection of 99mTc-SC may be a critical factor.

Also, some of the reduction in pain score recorded may be due to the technologist or physician bias secondary to knowing that topical lidocaine was being used in some patients. This bias is suggested by the result in group C (patients who had a discussion about using topical lidocaine but did not receive any topical lidocaine jelly prior to the injection of 99mTc-SC). Still, the pain score for group B was lower than that of group C (2.5 vs. 6.5, Table 1). The pain score for two women who received topical lidocaine jelly in the nuclear medicine department immediately prior to injection of 99mTc-SC, group (E), was 6. Finally, deep injections may lead to less reduction in pain associated with lymphoscintigraphy as the group without migration of radiopharmaceutical, group D, experienced pain similar to those without administration of topical lidocaine jelly, group A (8 vs. 8, Table 1).

## Conclusions

We conducted a review of the pilot trial administration of periareolar topical lidocaine to patients immediately after the completion of needle localization but prior to periareolar intradermal lymphoscintigraphy with 99mTc-SC. We found that patients experienced a substantial reduction in pain, and the effect appeared to be greater than if topical lidocaine was administered immediately prior to the injection. As such, we decided not to carry out a formal prospective blind study, and we now routinely administer topical lidocaine after needle localization to all patients who are scheduled to undergo periareolar intradermal injection of 99mTc-SC lymphoscintigraphy.
